# Effects of Fecal Microbiota Transplantation on Inflammatory Markers in Parkinson’s Disease: Exploratory Results from the EFFACE-PD Study

**DOI:** 10.3390/ijms27219924

**Published:** 2026-11-05

**Authors:** Monika Figura, Weronika Zając, Dagmara Otto-Ślusarczyk, Łukasz Milanowski, Julia Maya Nowak, Aleksandra Antoniak, Mateusz Kopczyński, Karol Sadowski, Łukasz Hołubiuk, Weronika Walęcik-Kot, Stanisław Szlufik, Jarosław Biliński, Marta Struga, Dariusz Koziorowski

**Affiliations:** 1 Department of Neurology, Faculty of Health Sciences, Medical University of Warsaw, Kondratowicza 8 St., 03-242 Warsaw, Poland; 2 Chair and Department of Biochemistry, Medical University of Warsaw, 02-097 Warsaw, Poland; dagmara.otto@wum.edu.pl (D.O.-Ś.);; 3 Faculty of Medicine, Medical University of Warsaw, 02-091 Warsaw, Polandkarol.sadowski@wum.edu.pl (K.S.); 4 Department of Gastroenterology, Masovian Brodnowski Hospital, 03-242 Warsaw, Poland; 5 Department of Hematology, Transplantation and Internal Medicine, Medical University of Warsaw, 02-097 Warsaw, Poland; 6 Human Biome Institute, 02-234 Warsaw, Poland

**Keywords:** inflammation, Parkinson’s disease, microbiota, cytokine

## Abstract

Inflammation and gut dysbiosis are increasingly recognized as important contributors to the pathophysiology of Parkinson’s disease (PD). Fecal microbiota transplantation (FMT) has emerged as a potential therapeutic strategy aimed at restoring gut microbial balance and modulating inflammatory pathways. The aim of the study was to evaluate the effect of single fecal microbiota transplantation on systemic and gut-related inflammatory biomarkers in patients with PD. This exploratory analysis was conducted as part of the randomized, double-blind, placebo-controlled EFFACE-PD study. Fifty-nine participants with PD were randomized to receive either donor-derived FMT or placebo. Serum levels of IL-18 and calprotectin were measured using ELISA before intervention and 3 months after the procedure. Ferritin, leukocyte count, neutrophils, lymphocytes, and neutrophil-to-lymphocyte ratio (NLR) were assessed at baseline and after twelve months. No statistically significant between-group differences in changes in inflammatory biomarker concentrations were observed. Broader inflammatory markers, including ferritin and leukocyte parameters, also did not differ significantly between groups. We conclude that single FMT did not induce significant changes in systemic inflammatory biomarkers in this exploratory cohort of PD patients. Larger studies with standardized inflammatory assessments are needed to clarify the immunomodulatory effects of FMT in PD.

## 1. Introduction

The role of inflammation in the pathogenesis and progression of Parkinson’s disease (PD) has been widely explored, highlighting its diagnostic and therapeutic potential. Neuroinflammation was first implicated in PD by McGeer et al., who demonstrated extensive microglial activation within the substantia nigra (SN) of persons with Parkinson’s disease (PwP) [1]. Subsequent development of the dual-hit hypothesis suggested that PD pathology may originate simultaneously in the olfactory bulb and enteric nervous system before propagating centrally through the vagus nerve and olfactory pathways [2]. This concept strengthened interest in the relationship between gut inflammation and PD pathogenesis. Gastrointestinal dysfunction, including constipation and delayed gastric emptying, frequently precedes motor symptoms by several years [3]. Moreover, elevated levels of inflammatory cytokines including IL-1β, IL-4, IL-6, IL-10, IL-18, tumor necrosis factor α (TNF-α), and transforming growth factor-β (TGF-β) have been identified in serum and cerebrospinal fluid of PwP [4,5,6]. Elevated calprotectin concentrations in serum and feces further support the presence of gut-related immune activation in PD [7]. Some studies reported an increase in PD incidence among people with inflammatory bowel diseases in Danish, Taiwanese and United States populations independently [8,9,10]. These associations have not been consistently replicated, and a causal relationship remains unproven. Additionally, Peter et al. indicate that anti-TNF-alpha therapy may decrease the risk of PD [10]. Several studies have demonstrated substantial alterations in gut microbiota composition in PwP compared with healthy individuals, with microbial changes correlating with disease severity and progression. These findings have positioned fecal microbiota transplantation (FMT) as a potential therapeutic strategy in PD. FMT is an established treatment for recurrent Clostridioides difficile infection and has shown promising effects on gastrointestinal symptoms and, in some reports, motor symptoms in PD [11,12]. Preclinical studies on PD animal models have shown that FMT may decrease both intestinal and central nervous system inflammation. Decreased expression of pro-inflammatory cytokines such as TNF-α, IL-1β, and IL-6 as well as attenuation of microglial and astrocyte activation was observed [13,14,15,16]. These effects are accompanied by restoration of gut microbiota homeostasis, enhancement of intestinal barrier integrity and preservation of nigrostriatal dopaminergic neurons [14]. These findings support the hypothesis that FMT-based modulation of the gut–brain axis is a potential mechanism of neuroprotection in PD. However, confirmation of these effects in PwP remains lacking.

The primary clinical outcomes of the EFFACE-PD trial, including motor and non-motor symptoms, gastrointestinal function, and quality of life, have been reported previously [17]. The aim of this exploratory analysis of the samples collected during EFFACE-PD trial is to assess whether single FMT may affect systemic inflammatory biomarker expression in PwP [17].

## 2. Results

The exploratory analysis was based on the samples obtained from 59 participants randomized in the EFFACE-PD trial [17]. The number of available samples varied by biomarker and follow-up time point. Table 1 summarizes baseline demographic data of the patients. Because of missing samples, inflammatory biomarker measurements at baseline and 3 months were available for only a subset of participants.

No statistically significant between-group differences in changes in the assessed circulating inflammatory biomarkers were observed at 3 months. The change in IL-18 did not differ significantly between the FMT and placebo groups (*p* = 0.955). Similarly, no significant between-group differences were observed for calprotectin (*p* = 0.536). Within-group analyses also showed no statistically significant changes in IL-18 or calprotectin concentrations between baseline and the 3-month follow-up in either group. IL-18 concentrations numerically decreased in both groups, whereas calprotectin demonstrated substantial interindividual variability. Table 2. summarizes the results for baseline and 3-month follow-up.

At 12 months, no statistically significant between-group differences in change were observed for ferritin (*p* = 0.673), leukocyte count (*p* = 0.351), neutrophil count (*p* = 0.864), lymphocyte count (*p* = 0.339), or neutrophil-to-lymphocyte ratio (NLR) (*p* = 0.821). Within-group comparisons revealed no statistically significant changes in any of these parameters. Ferritin concentrations and peripheral blood inflammatory indices therefore remained broadly stable over the 12-month follow-up. All the results are summarized in Table 3.

## 3. Discussion

A growing body of evidence supports the role of inflammation and gut dysbiosis in PD pathophysiology. Alterations in intestinal permeability and microbiota composition may contribute to systemic inflammation, neuroimmune activation, and α-synuclein pathology. FMT therefore represents a potentially disease-modifying therapeutic approach. While previous studies have primarily focused on the clinical effects of FMT in PD, its impact on systemic inflammatory processes remains poorly understood. Therefore, this analysis aimed to investigate whether FMT induces measurable changes in selected inflammatory markers, potentially providing further insight into the biological mechanisms underlying this intervention. In this study in addition to inflammatory cytokines, we assessed a broader panel of markers of systemic inflammation in this patient cohort, including serum ferritin, total white blood cell count, and differential leukocyte counts. We conclude that a single colonoscopy-delivered FMT was not associated with a clear or statistically significant anti-inflammatory effect. This finding contrasts with the relatively consistent anti-inflammatory effects reported in preclinical models of PD [13,14,15]. Several factors may account for the discrepancy between these experimental findings and our observations. First, toxin-induced animal models represent relatively homogeneous experimental systems characterized by pronounced and acutely induced inflammatory responses, whereas inflammation in human PD is likely to be substantially more heterogeneous in its magnitude, temporal profile, and underlying mechanisms. Indeed, human studies demonstrate considerable interindividual variability in inflammatory markers. Although alterations in circulating pro- and anti-inflammatory cytokines, including IL-6 and TNF-α, have been reported in PD, the inflammatory profile appears to be heterogeneous, with individual cytokines showing distinct associations with demographic and clinical characteristics and disease severity [18]. Thus, an anti-inflammatory response to microbiota modulation may occur primarily in a subgroup of patients with a pre-existing inflammatory phenotype and may consequently be diluted in analyses performed at the whole-group level [19].

Second, changes induced by FMT at the intestinal level may not necessarily be reflected by parallel changes in circulating inflammatory markers. Evidence from PD cohorts indicates the presence of intestinal inflammation, including elevated fecal calprotectin and alterations in short-chain fatty acid concentrations and intestinal barrier-related markers [20]. Importantly, studies simultaneously assessing intestinal and systemic markers indicate that these compartments may not closely correspond, as demonstrated by a study by Dumitrescu et al. [21]. Therefore, the absence of significant changes in serum cytokines, ferritin, or circulating leukocyte populations in our study does not necessarily exclude a localized immunomodulatory effect of FMT within the intestinal mucosa.

A study by Leta et al. of four-strain probiotic treatment indicated that an intervention aimed at modulating gut microbiota in PwP may result in a decrease in inflammatory cytokines, namely TNF-α and IL-6 [22]. Similarly, in a study by Ramadan et al. assessing the results of treatment with a synbiotic (*Lactobacillus acidophilus* probiotic and inulin prebiotic), the symbiotic group exhibited significantly lower serum levels of TNF-α and oxidative stress marker MDA, and significantly higher levels of the neuroprotective factor BDNF [23]. Both these interventions were administered repeatedly. We therefore hypothesize, that anti-inflammatory effect of FMT could only be achieved after consolidation obtained with repeated doses of FMT administered orally. Randomized trials of single FMT performed by Bruggeman and Scheperjans did not include data on inflammatory response to the procedure [24,25].

Gut-specific immune modulation may not be fully reflected by systemic cytokine measurements. Moreover, inflammatory status in PD is influenced by multiple confounding factors, including age, comorbidities, disease duration, diet, and physical activity [20,26]. This study has several limitations, including the small number of samples available for cytokine analysis and the exploratory nature of the investigation. These results should therefore be interpreted with caution and need further validation. We assessed lymphocyte count, WBC, and NLR one year after the intervention (FMT). Therefore, even if transient changes had occurred earlier, they may no longer have been detectable at the one-year follow-up.

## 4. Materials and Methods

This study represents an exploratory analysis of the EFFACE-PD trial, a parallel-group, randomized, double-blind, placebo-controlled study registered at ClinicalTrials.gov (NCT05204641) [17]. Fifty-nine participants with PD were enrolled, including 28 patients receiving donor-derived FMT and 31 receiving placebo (auto-FMT). FMT was performed via colonoscopy. The FMT preparation consisted of 60 g of stool obtained from healthy donors, suspended in 200 mL of 0.9% sodium chloride solution containing 10% glycerol and stored frozen. An identical preparation was used for the placebo group, containing each participant’s own stool (autologous FMT) collected before antibiotic treatment and colonoscopy. The details of the procedure and product characteristics are available in the paper by Figura et al. [17].

All participants provided written informed consent. The study protocol was approved by the Ethics Committee of the Medical University of Warsaw (KB/129/2021) with further amendments (KB/108/A2021 and KB/13/A2022) and conducted in accordance with the Declaration of Helsinki. Blood samples were collected before FMT and during follow-up visits. Five milliliters of whole blood were obtained before the intervention and three months post-intervention for cytokine analysis. Plasma samples were collected following centrifugation at 4 °C and stored at −80 °C until analysis.

Venous blood samples for complete blood count and ferritin were collected before the procedure and 12 months after. For leukocytes, the blood was collected into EDTA-containing tubes gently inverted to ensure adequate mixing with the anticoagulant. For serum ferritin measurements, blood was drawn into serum-separating tubes with gel and clot activator, allowed to clot at room temperature for 30 min, and subsequently centrifuged at 2000× *g* for 10 min at 4 °C. The number of leukocytes, lymphocytes and neutrophils was determined using Sysmex XN 1000 automated hematology analyzer (Kobe, Japan). Serum ferritin was measured by electrochemiluminescence immunoassay (ECLIA) using an Abbott–Alinity analyzer (Irving, TX, USA). All analyses were performed in a certified clinical laboratory in accordance with the manufacturer’s protocols and standard operating procedures.

### Enzyme-Linked Immunosorbent Assay

Plasma concentrations of calprotectin and IL-18 were measured using commercial ELISA kits (Human Calprotectin ELISA kit, Abcam, Cambridge, UK; Human Total IL-18 ELISA kit, Proteintech, Rosemont, IL, USA) according to the manufacturers’ instructions.

Next, 100 μL of standards, controls, and samples were added to the wells on pre-coated 96-well plates and incubated for 2 h at 37 °C. Plates were then washed four times with 1× wash buffer. Subsequently, 100 μL of detection antibody was added to each well and incubated for 1 h at 37 °C, followed by washing. Next, 100 μL of streptavidin–HRP solution was added and incubated for 40 min at 37 °C, followed by another wash step. For signal development, 100 μL of TMB substrate was added and incubated in the dark for 20 min. The reaction was stopped with 100 μL of stop solution. Absorbance was measured immediately at 450 nm and at 630 using a microplate reader (Agilent BioTek Synergy H1 Multimode Reader, Winooski, VT, USA).

Concentrations were calculated from standard curves generated using a four-parameter logistic (4-PL) model after subtraction of blank values. Same procedure was applied for determination of human calprotectin concentration using ELISA kit. The samples were diluted 4000 times with solution diluent. Absorbance was measured only at 450 nm using a microplate reader.

Sensitivity and assay range were as follows: S: IL-18 = 0.3 pg/mL, calprotectin = 35 pg/mL; AR: IL-18 = 15.6–1000 pg/mL, calprotectin = 32.77–8000 pg/mL.

Statistical analyses were performed using STATISTICA v. 13.5 software (TIBCO Software Inc., Palo Alto, CA, USA). Normality of continuous variables was assessed using the Shapiro–Wilk test. Because several inflammatory biomarkers showed non-normal distributions and substantial interindividual variability, particularly in the cytokine subgroup, continuous variables are presented as median and interquartile range (IQR).

Within-group changes between baseline and follow-up were assessed using the Wilcoxon signed-rank test for paired samples. For each biomarker, individual change was calculated as the baseline value minus the corresponding follow-up value; therefore, positive values indicate a decrease, and negative values indicate an increase over time. Between-group differences in change from baseline between the FMT and placebo groups were assessed using the Mann–Whitney U test. Exact two-sided *p* values were used for comparisons involving the small cytokine subgroups. All statistical tests were two-sided, and a *p* value < 0.05 was considered statistically significant.

## 5. Conclusions

In conclusion, single FMT did not produce statistically significant changes in systemic inflammatory biomarkers in PwP. However, data from other studies suggest an anti-inflammatory potential of microbiota- directed interventions [22,23]. We hypothesize that repeated dosing of FMT could be of benefit to achieve a sustained anti-inflammatory effect. Future studies should incorporate larger cohorts, longitudinal inflammatory profiling, and fecal biomarkers to better characterize the immunological effects of FMT in PD. Further large-scale studies are required to clarify the immunomodulatory role of FMT in PD.

## Figures and Tables

**Table 1:** Baseline sociodemographic and clinical factors, and symptoms of PD in patients (N  =  59) classified by the Intervention Group. * LEDD and UPDRS III are presented as estimated marginal mean and 95% CI. Age and symptom duration are presented as median and IQR.

Characteristic	Intervention Group	
	FMT	Placebo
*N*	28	31
Females (%)	13 (46%)	17 (55%)
Age (yr)	65.0 (58.0, 69.3)	63.0 (56.5, 67.0)
Symptom duration, (yr)	5.0 (2.8, 7.0)	6.0 (2.0, 7.5)
* LEDD at baseline	796.0 [625.1 to 966.9]	742 [562.7 to 921.3]
* UPDRS III OFF at baseline (95%CI)	21.5 [17.6 to 25.4]	22.5 [18.4 to 26.6]

**Table 2:** Changes in circulating inflammatory biomarkers from baseline to 3 months after fecal microbiota transplantation.

Biomarker/Parameter	Time Point	FMT, Median [IQR] (n)	Placebo, Median [IQR] (n)	Between-Group *p* for Change
IL-18 (pg/mL)	Baseline	206.96 [81.88–411.94] (n = 8)	195.33 [93.33–588.12] (n = 7)	
	3 months	70.88 [53.86–136.12] (n = 8)	165.07 [51.38–282.79] (n = 7)	
	Change	115.3 [−59.0–357.2] (n = 8)	78.30 [−140.3–772.5] (n = 7)	0.955
	Within-group p ^1^	0.208	0.310	
Calprotectin (ng/mL)	Baseline	28.24 [11.63–60.32] (n = 8)	68.75 [25.33–70.42] (n = 7)	
	3 months	38.86 [26.12–59.42] (n = 8)	67.08 [43.37–102.50] (n = 7)	
	Change	−2.7 [−20.0–12.3] (n = 8)	−25.0 [−96.9–49.9] (n = 7)	0.536
	Within-group p ^1^	1.000	0.499	

Data are presented as median [interquartile range (IQR)]. Change was calculated as baseline minus the 3-month value; therefore, positive values indicate a decrease and negative values indicate an increase from baseline. ^1^ Within-group *p* values were calculated using the Wilcoxon signed-rank test for paired samples. Between-group differences in change from baseline were assessed using the Mann–Whitney U test; exact two-sided *p* values are reported. FMT, fecal microbiota transplantation; IL-18, interleukin-18.

**Table 3:** Changes in ferritin and peripheral blood inflammatory indices from baseline to 12 months after fecal microbiota transplantation.

Biomarker/Parameter	Time Point	FMT, Median [IQR] (n)	Placebo, Median [IQR] (n)	Between-Group *p* for Change
Ferritin (ng/mL)	Baseline	124.0 [84.2–178.85] (n = 27)	95.7 [50.65–133.35] (n = 31)	
	12 months	100.6 [66.17–133.25] (n = 24)	72.35 [39.82–116.08] (n = 28)	
	Change	1.0 [−2.0–35.0] (n = 23)	3.30 [−4.0–19.3] (n = 28)	0.673
	Within-group p ^1^	0.067	0.143	
Leukocytes (G/L)	Baseline	5.63 [4.89–6.29] (n = 27)	5.97 [5.58–7.08] (n = 31)	
	12 months	5.60 [4.52–7.22] (n = 27)	6.07 [5.28–7.12] (n = 29)	
	Change	−0.3 [−1.0–0.5] (n = 25)	0.05 [−0.8–0.7] (n = 30)	0.351
	Within-group p ^1^	0.316	0.982	
Neutrophils (G/L)	Baseline	3.21 [2.62–3.81] (n = 27)	3.48 [3.00–4.105] (n = 31)	
	12 months	2.96 [2.31–4.66] (n = 27)	3.62 [2.94–4.56] (n = 29)	
	Change	0.1 [−1.0–0.6] (n = 26)	0.02 [−0.9–0.6] (n = 30)	0.864
	Within-group p ^1^	0.809	0.545	
Lymphocytes (G/L)	Baseline	1.68 [1.51–2.05] (n = 27)	2.11 [1.765–2.245] (n = 31)	
	12 months	1.71 [1.21–2.115] (n = 27)	1.83 [1.64–2.18] (n = 29)	
	Change	−0.0 [−0.0–0.4] (n = 26)	0.13 [−0.1–0.4] (n = 30)	0.339
	Within-group p ^1^	0.859	0.086	
NLR	Baseline	1.79 [1.48–2.30] (n = 27)	1.77 [1.50–2.15] (n = 31)	
	12 months	1.93 [1.27–2.48] (n = 27)	2.14 [1.39–2.61] (n = 29)	
	Change	−0.1 [−1.0–0.5] (n = 26)	−0.12 [−0.6–0.4] (n = 29)	0.821
	Within-group p ^1^	0.551	0.222	

Data are presented as median [interquartile range (IQR)]. Change was calculated as baseline minus the 12-month value; therefore, positive values indicate a decrease, and negative values indicate an increase from baseline. ^1^ Within-group *p* values were calculated using the Wilcoxon signed-rank test for paired samples. Between-group differences in change from baseline were assessed using the Mann–Whitney U test. The number of available observations varied across biomarkers and time points because of samples missing. FMT, fecal microbiota transplantation; NLR, neutrophil-to-lymphocyte ratio.

## Data Availability

The data supporting the findings of this study are available from the corresponding author upon reasonable request.
